# *CRTC1::TRIM11* Cutaneous Tumors With Atypia: Melanoma Mimicry, Aggressive Potential, and Methylation Classifier Limitations

**DOI:** 10.1002/gcc.70127

**Published:** 2026-04-21

**Authors:** Bethany Batson, Arivarasan Karunamurthy, Azfar Neyaz, John M. Skaugen, Steven D. Billings, Karen Fritchie, Ivy John

**Affiliations:** ^1^ Department of Pathology University of Pittsburgh Pittsburgh Pennsylvania USA; ^2^ Department of Dermatology University of Pittsburgh Pittsburgh Pennsylvania USA; ^3^ Department of Pathology and Laboratory Medicine Cleveland Clinic Cleveland Ohio USA

**Keywords:** clear cell sarcoma, *CRTC1::TRIM11* cutaneous tumor, malignant, melanoma, methylation, *TERT* promoter mutation

## Abstract

*CRTC1::TRIM11* cutaneous tumors are an emerging subset of MITF pathway–activated neoplasms that typically present as dermal nodules and can closely mimic clear cell sarcoma or malignant melanoma. Most reported tumors behave in an indolent manner, yet rare malignant cases have been documented. We report three additional *CRTC1::TRIM11* cutaneous tumors with atypical features, including two patients with nodal or visceral disease at presentation. Histologically, all three tumors showed marked cytologic atypia with prominent nucleoli, and the usual nested and short‐fascicular architecture was largely replaced by broad sheet‐like growth, raising concern for melanoma. The tumors expressed SOX10 (diffuse) and S100 protein (patchy) with focal to absent expression of Melan‐A and HMB45. PRAME was negative. *CRTC1::TRIM11* was confirmed in all tumors. Two tumors harbored *TERT* promoter mutations, and one showed additional low‐level copy‐number gains of 1q, 8q, and 12q with 14q loss. In one case, methylation profiling yielded a clear cell sarcoma score of 0.885, just below the confidence threshold, likely due to shared CREB–MITF pathway activation and the lack of a dedicated *CRTC1::TRIM11* reference class. A literature review identified nine previously reported metastatic *CRTC1::TRIM11* cutaneous tumors. When combined with our series, extremities were the predominant primary site, and metastases most often involved regional lymph nodes and the lung. Fusion status remains the molecular gold standard, while secondary events such as *TERT* promoter mutations and 8q gains may contribute to aggressive behavior in a subset of tumors.

## 1 Introduction

*CRTC1::TRIM11* cutaneous tumors are an emerging subset of MITF‐pathway activated neoplasms that present in the dermis as amelanotic nodules and often mimic clear cell sarcoma or melanoma. Most reported tumors follow an indolent course; nonetheless, nine cases with nodal or distant metastasis have now been described. We describe three additional *CRTC1::TRIM11* cutaneous tumors with atypical features, two of which showed nodal or visceral disease at presentation. We outline their differential diagnosis with an emphasis on melanoma, discuss emerging molecular features linked to aggressive behavior, and address why sarcoma methylation classifiers may misclassify these tumors as clear cell sarcoma.

**FIGURE 1:**
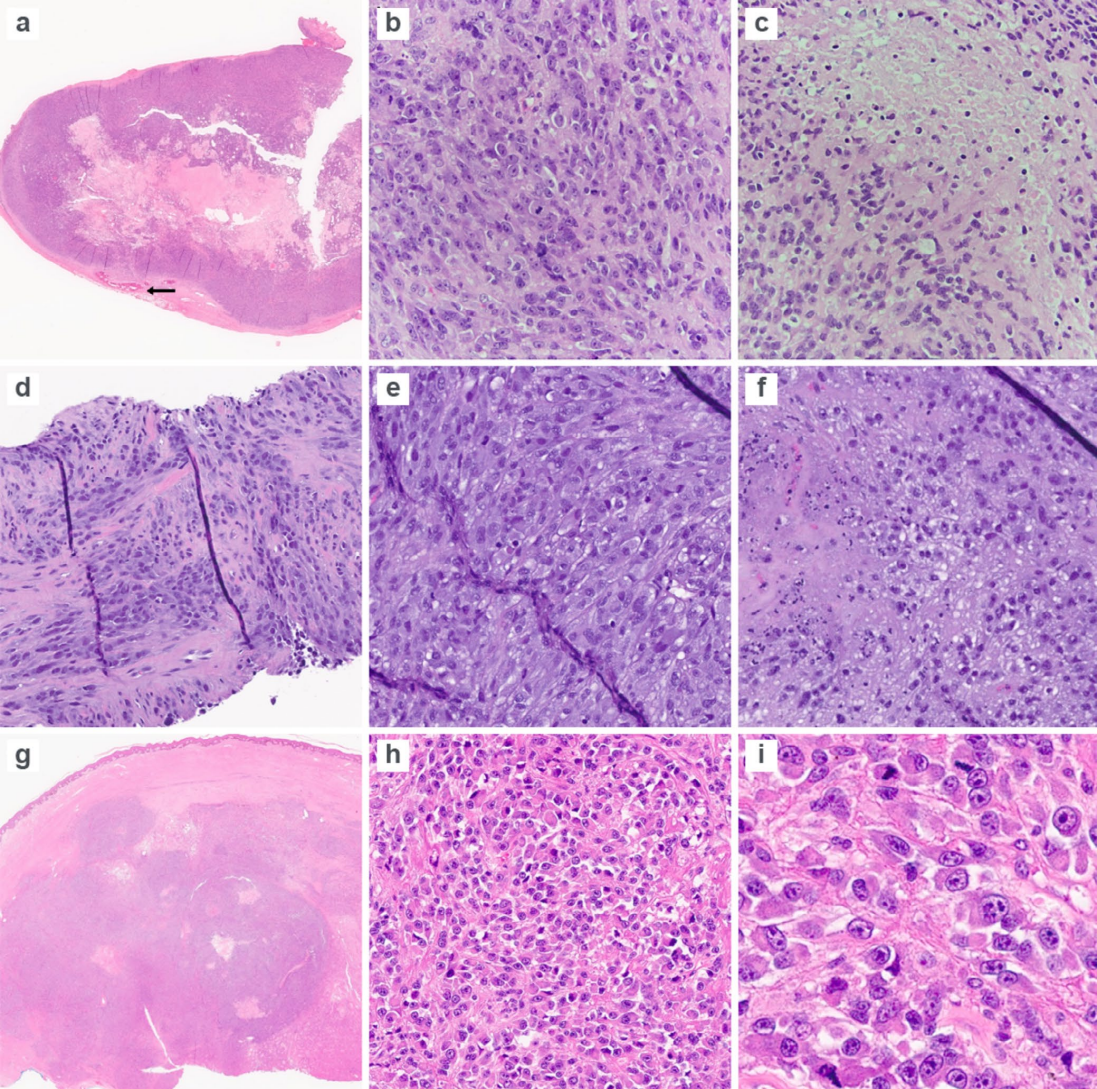
Case 1: The axillary lymph node is largely replaced by tumor, with a peripheral focus of osseous metaplasia (arrow, a). The neoplasm is composed of sheets of epithelioid cells with vesicular nuclei, prominent nucleoli, marked cytologic atypia, and brisk mitotic activity (b). Focal areas of necrosis are present (c). Case 2: Core needle biopsy demonstrating bundles and sheets of atypical epithelioid cells with amphophilic cytoplasm (d and e). Focal areas of necrosis are present (f). Case 3: Lobulated tumor centered in the dermis composed of epithelioid to plasmacytoid cells with eosinophilic cytoplasm (g and h). The tumor shows marked cytologic atypia and brisk mitotic activity (i).

Case 1 A 64‐year‐old woman presented with a 7 cm fungating left arm mass and palpable ipsilateral axillary lymphadenopathy. An excision of the axillary lymph node and a punch biopsy of the skin overlying the primary mass were performed. Histologic examination of the lymph node showed that it was largely replaced by tumor composed of nests and sheets of non‐pigmented spindle to epithelioid cells with prominent nucleoli, pale eosinophilic cytoplasm, marked cytologic atypia, brisk mitoses, patchy necrosis, hyalinized vessels, and peripheral osseous metaplasia. In contrast, the punch biopsy demonstrated more characteristic features, including spindled cells arranged in short bundles without significant atypia or mitotic activity (Figure 1a–c). Immunohistochemistry demonstrated diffuse SOX10 and MITF positivity with patchy S100 protein expression, retained INI‐1 and H3K27me3 expression, and negativity for Melan‐A, PRAME, tyrosinase, HMB45, desmin, SMA, AE1/AE3, and CD34. Fluorescence in situ hybridization (FISH) was negative for rearrangements involving *EWSR1* and *FUS*. Targeted next‐generation sequencing (Oncomine v3, Thermo Fischer) identified a *TERT* promoter mutation with low‐level gains of 1q, 8q, and 12q, a 14q loss, no *BRAF/NRAS/NF1* mutations, microsatellite stability, and a low tumor mutational burden (< 1/Mb). Whole‐transcriptome RNA sequencing identified a *CRTC1::TRIM11* fusion involving exon 1 of *CRTC1* and exon 2 of *TRIM11*. DNA methylation profiling (sarcoma classifier v12.3) yielded a clear cell sarcoma score of 0.885, just below the high‐confidence threshold.

Case 2 A 70‐year‐old woman presented with a 3 cm lesion on the leg and a concurrent lung mass. Multiple core needle biopsies of the leg lesion were obtained. Histologically, the neoplasm consisted of spindled to epithelioid cells, often showing significant cytologic atypia and amphophilic cytoplasm, arranged in short bundles and sheets. Scattered mitotic figures, including atypical forms (up to 3 per 10 high‐power fields), and focal necrosis were present (Figure 1d–f). Immunohistochemically, the tumor cells were diffusely and strongly positive for SOX10 with weak, patchy S100 protein expression. Stains for cytokeratins (CAM5.2, CK7, CK20), PAX8, CDX2, TTF‐1, CD31, CD34, SMA, desmin, Melan‐A, and PRAME were negative. CC‐SIGN Solid Tumor Gene Fusion NGS Panel confirmed a *CRTC1::TRIM11* fusion. DNA sequencing was not performed. The lung lesion was not biopsied but was clinically presumed to represent metastatic disease. Clinical follow‐up was not available for this case, which was received in consultation.

Case 3 A 74‐year‐old man presented with a 4.8 cm lesion on the leg. Histologically, the tumor was dermally centered and multilobulated, composed of neoplastic cells arranged in nests and sheets. The cells were spindle to epithelioid, with some showing plasmacytoid features, and had vesicular nuclei, prominent nucleoli, and eosinophilic cytoplasm. A subset displayed marked cytologic atypia. Mitotic activity was brisk, reaching up to 11 mitoses per high‐power field, and areas of necrosis were noted (Figure 1g–i). Immunohistochemically, the tumor showed diffuse SOX10 positivity with patchy S100 protein and rare Melan‐A–positive cells. The cells were negative for p40, TTF‐1, PAX8, GATA3, CDX2, PSA, NKX3.1, synaptophysin, ERG, CK AE1/AE3, CK7, CK20, CK8/18, and CK5/6. Molecular studies, including CC‐SIGN Solid Tumor Gene Fusion NGS Panel and Targeted next‐generation sequencing, confirmed a *CRTC1::TRIM11* fusion along with a *TERT* promoter mutation and a tumor mutational burden of three mutations/Mb.

## 2 Discussion

*CRTC1::TRIM11* cutaneous tumors are dermal‐based neoplasms defined by an in‐frame fusion joining *CRTC1* exon 1 to *TRIM11* exon 2. The initial report of five cases described well‐circumscribed intradermal nodules composed of epithelioid to spindled melanocytic cells with diffuse SOX10 and MITF expression, variable S100 protein expression, and inconsistent Melan‐A and HMB45 immunoreactivity [1]. Clinical follow‐up in that cohort was limited, although all patients remained disease‐free. The largest multi‐institutional study of 41 molecularly confirmed tumors reinforced the reproducibility of the morphologic and immunophenotypic profile, while documenting one local recurrence and one regional nodal metastasis, establishing that, despite an overall indolent course, a malignant subset exists [2].

To better characterize the malignant potential of this tumor type, we reviewed the literature and identified nine metastatic *CRTC1::TRIM11* cutaneous tumors among 61 reported cases (15%) [2, 3, 4, 5, 6, 7, 8, 9, 10, 11]. In our series, two additional cases showed evidence of metastatic disease, while a third demonstrated aggressive histologic and molecular features suggestive of malignant potential, although metastasis could not be confirmed due to limited follow‐up. Including our cases, 10 of 12 tumors (83%) arose on the extremities, with the remaining two involving the upper back and the nasal mucosa. The age at presentation ranged widely from 5 to 75 years (mean 39.5 years), and there was a female predominance (7/11; 64%) as compared to male (4/11; 36%). Tumor size varied from 0.6 to 7.0 cm (average 3.7 cm). Histologically, most tumors were centered in the dermis or subcutis (9 of 10, 90%), with epidermal involvement documented in only one case (10%). Necrosis was present in four cases (36%), lymphovascular invasion in two cases (18%), and cytologic atypia in six cases (55%). Five of the eleven cases (45%) had evidence of metastases at time of presentation, with 4 cases developing metastasis less than 2 years after diagnosis, and 2 cases with late metastases, 11 and 13 years after presentation. Metastases most frequently involved regional lymph nodes, lungs, and soft tissue. Follow‐up ranged from 7 to 156 months (median 29 months). Among the nine cases with available outcome data, six patients were alive with disease (67%), two were alive without disease (22%), and one had died of disease (11%) (Table 1).

**TABLE 1:** Clinicopathologic features of *CRTC1::TRIM11* cutaneous tumors with high‐risk features and metastasis.

Case	Primary site	Sex	Age at dx.	Size (cm)	Atypia	Necrosis	Mitoses	LVI	Recurrence (Y/N)	Time to recurrence (months)	Metastasis (Y/N)	Time to metastasis (months)	Status	Length of follow‐up (months)	Molecular features
1 [3]	Right arm	F	31	5.13	N	N	N		Y	156	Y	156	Alive with metastatic disease	156	*CRTC1::TRIM11*
2 [4]	Right Hip	M	30	1	Y	N	8 per 10 HPF		N	N/A	Y	18, 26, 30, 36	Alive with progressive nodal and lung metastases	36	*CRTC1:: TRIM11* *TERT* promoter mutation c.124 C>T *MET* and *FGFR1* amplification
3 [2]	Hand	NR		NR	N	N	N		N	N/A	Y	13	Alive with nodal metastases	13	*CRTC1::TRIM11*
4 [5, 6, 7]	Right upper arm	F	5	0.6	Y	N	10/mm^2^		Y	19	Y	19	Alive with stable metastatic disease on dual immunotherapy	29	*CRTC1::TRIM11* Decreased *CDKN2A* RNA expression
5 [8]	Right nasal mucosa	F	23	NR	N	N	< 5 per 10 HPF		Y	132	Y	132	Alive with nodal, soft tissue, and lung metastasis undergoing chemoradiation	132	*CRTC1::TRIM11* *TERT* promoter mutation c.146C>T
6 [9]	Right fifth toe	M	75	2.5	N	N	12/mm^2^	Y	Y	2	Y	5 and 8	Alive with nodal and radiologically suspected lung metastases despite immunotherapy	8	*CRTC1::TRIM11* Germline *BRCA2* variant
7 [10]	Right posterior thigh	F	29	2.5	N	N	Rare	Y	N	N/A	Y	0	Alive without disease	7	
8 [11]	Upper back	F	17	6.4	Y	Y	7/mm^2^		N	N/A	Y	0	Died of disease	18	*CRTC1::TRIM11* *BRAF* intronic deletion (1‐14) Germline *APC* mutation c.3921 T>A Tumor ploidy:3.38 and 8q gain TMB:0.98
9 [7]	Right Ankle	M	17	NR	NR	NR	8/mm^2^		N	N/A	Y	0	Alive without evidence of disease	48	*CRTC1::TRIM11*
10	Left arm	F	64	7.0	Y	Y	15 per 10 HPF		N	N/A	Y	0	NR	NR	*CRTC1::TRIM11* *TERT* promoter mutation c.124C>T 14q loss and low level 1q,8q,12q gains
11	Leg	F	70	3.0	Y	Y	3 per 10 HPF		N	N/A	Y^a^	0	NR	NR	*CRTC1::TRIM11*
12	Leg	M	74	4.8	Y	Y	11 per 10 HPF		N	N/A	N	N/A	NR	NR	*CRTC1::TRIM11* *TERT* promoter mutation c.124 C>T TMB: 3

*Note:* ^a^Presumed. Including the three additional cases, a total of 64 cases of *CRTC1::TRIM11* rearranged cutaneous tumors have been reported, of which 11 showed metastatic behavior representing 17% of cases.

Abbreviations: F: Female, M: Male, N/A: Not applicable, NR: Not reported.

Although *CRTC1::TRIM11* cutaneous tumors typically grow as well‐circumscribed, dermal nodules composed of short intersecting fascicles and nests of relatively uniform epithelioid‐to‐spindle cells, our cases departed from this pattern. All three tumors showed marked cytologic atypia with prominent nucleoli, and the characteristic architecture was largely lost and replaced by a broad, sheet‐like growth. In this setting, particularly when nodal or pulmonary disease is present, melanoma becomes the immediate diagnostic consideration. One feature that still argues against a primary cutaneous melanoma is the absence of a true intraepidermal component. *CRTC1::TRIM11* cutaneous tumors almost always spare the epidermis even when they abut it, whereas most primary melanomas demonstrate an in situ or junctional component. The caveat, however, is that cutaneous melanoma metastases can also lack a junctional proliferation, so this feature is helpful but not definitive.

Immunohistochemistry may offer supportive clues but is not conclusive. SOX10 expression is typically diffuse in both *CRTC1::TRIM11* cutaneous tumors and melanoma. In *CRTC1::TRIM11* cutaneous tumors, however, S100 protein expression is often patchy, and Melan‐A and HMB45 immunoreactivity is frequently focal or negative, whereas melanomas more commonly show diffuse staining for these markers. PRAME is frequently expressed in melanoma and usually absent in fusion‐driven MITF‐pathway tumors. Therefore, a positive result can be helpful, although a negative PRAME stain does not exclude melanoma [9, 11, 12, 13]. In keeping with these observations, PRAME was negative in both of the cases we tested.

Ultimately, reliable distinction between these tumors depends on molecular confirmation. Melanomas, unlike *CRTC1::TRIM11* cutaneous tumors, commonly harbor mutations in *BRAF*, *NRAS*, *KIT*, or *NF1* and often exhibit a UV mutational signature and higher tumor mutational burden [14]. Additionally, they do not harbor *CRTC1::TRIM11* fusions.

Clear cell sarcoma is another histologic mimic of *CRTC1::TRIM11* cutaneous tumors. Both are SOX10‐ and S100 protein‐positive and show a nested or short‐fascicular growth of epithelioid to spindle cells with prominent nucleoli. However, there are several distinguishing features. Clear cell sarcoma generally arises in deeper soft tissues with infiltrative borders, may contain characteristic wreath‐like multinucleated giant cells, and is defined molecularly by *EWSR1::ATF1* or *EWSR1::CREB1* fusions.

Finally, several other recently described fusion‐associated cutaneous neoplasms with melanocytic differentiation should be considered in the differential diagnosis. *ACTIN::MITF*‐rearranged tumors typically show epithelioid cells with clear cytoplasm arranged in a checkerboard‐like growth pattern and are often SOX10‐negative, an immunophenotype that contrasts with the diffuse SOX10 expression seen in *CRTC1::TRIM11* cutaneous tumors [15]. *MITF::CREM*‐rearranged tumors are dermal‐based lesions with an infiltrative growth pattern and can be sheet‐forming with marked atypia and brisk mitotic activity. They are SOX10‐ and S100‐positive, and distinction from *CRTC1::TRIM11* tumors often requires fusion testing [16]. *MED15::ATF1* rearranged tumors are rare, dermal, wedge‐shaped to nodular neoplasm, usually arising in children and adolescents, composed of nests and fascicles of ovoid to round cells, at times with spitzoid features. Like *CRTC1::TRIM11* cutaneous tumors, they are SOX10‐ and MITF‐positive with limited or absent S100, HMB45, and Melan A expression, and definitive separation therefore relies on fusion testing [7, 17].

Although *CRTC1::TRIM11* is the defining driver, emerging evidence suggests that additional second‐hit events may contribute to tumor progression (Table 1). Telomerase reactivation is the best‐documented example. In one early‐metastasizing *CRTC1::TRIM11* cutaneous tumor, next‐generation sequencing identified a *TERT* promoter hotspot mutation, c.‐124C>T, together with *MET* and *FGFR1* co‐amplifications [4]. Another case of *CRTC1::TRIM11* cutaneous tumor with metastatic disease also demonstrated *TERT* promoter c.‐124C>T [9]. Our series adds two *TERT*‐promoter mutant tumors, including one with nodal involvement, further supporting a role for telomerase activation in driving aggressive behavior in a subset of *CRTC1::TRIM11* cutaneous tumors. Additional copy‐number alterations also likely play a role in their progression. Case 1 showed low‐level copy number gains involving 1q, 8q, and 12q chromosomal regions and a 14q copy number loss in the setting of very low tumor mutational burden. Similarly, Beatson et al. reported a malignant *CRTC1::TRIM11* cutaneous tumor in a 17‐year‐old girl that metastasized to lymph nodes, lung and soft tissue and was fatal 18 months after presentation [11]. Comprehensive genomic profiling in that case revealed multiple additional aberrations, including an intragenic *BRAF* deletion and widespread copy‐number gains with high‐level 8q amplification involving *MYC* and *AGO2*, in the setting of very low tumor mutational burden and absence of a *TERT* promoter mutation [11]. In contrast, among the non‐metastatic *CRTC1::TRIM11* cutaneous tumors, there are only two cases with copy number changes involving gains in chromosome 7 [1]. Of note, clear cell sarcoma, which shares CREB–MITF pathway activation, provides a useful point of comparison and frequently harbors chromosome 8q gains, often involving the *MYC* locus [18, 19]. Additionally, *TERT* promoter hotspot mutations have been reported as recurrent secondary alterations in clear cell sarcoma and have been associated with inferior survival, supporting a role for telomerase activation as a potential progression event in a subset of cases [20].

Our first case highlights the need for caution when interpreting methylation profiling results in the evaluation of uncommon melanocytic and soft tissue tumors in contemporary practice. In Case 1, the sarcoma methylation classifier (v12.3) returned a calibrated score of 0.885 for the clear cell sarcoma class, just below the 0.90 threshold required for a confident classification. Two factors likely contributed to this result. First, clear cell sarcoma is defined by *EWSR1::ATF1* or *EWSR1::CREB1* fusions, which activate CREB‐family–mediated transcription and are associated with melanocytic differentiation and MITF expression, whereas *CRTC1::TRIM11* cutaneous tumors involve a fusion of *CRTC1*, a CREB‐regulated transcription coactivator, and likewise show MITF‐associated melanocytic differentiation [17, 21, 22]. This shared CREB‐linked transcriptional biology may lead to overlapping gene‐expression patterns and, in turn, partially similar DNA‐methylation profiles. Second, public sarcoma methylation classifiers are trained on large, curated reference cohorts, and rare or newly recognized entities with limited representation tend to be projected onto the closest well‐represented class [23, 24, 25] This is a recognized limitation of methylation classifiers and is particularly relevant here because current training sets do not yet include a dedicated *CRTC1::TRIM11* cutaneous tumor reference category. As *CRTC1::TRIM11* cutaneous tumors are further characterized, future iterations of diagnostic classifiers may include a dedicated reference category, thereby enhancing diagnostic accuracy.

In summary, *CRTC1::TRIM11* cutaneous tumors with atypical features may closely mimic malignant melanoma. Molecular studies are required for its confident distinction. Emerging evidence suggests that additional genomic events, including *TERT* promoter mutations and 8q (*MYC*) amplification, likely contribute to aggressive behavior in a subset of tumors. Because *CRTC1::TRIM11* cutaneous tumors and clear cell sarcoma share CREB–MITF pathway activation, methylation classifiers can misclassify these rare tumors as part of the clear cell sarcoma group, particularly when training sets lack dedicated *CRTC1::TRIM11* cutaneous tumor references. Until classifier coverage improves, orthogonal confirmation of the gene fusion should remain the molecular gold standard for diagnosis.

## Funding

The authors have nothing to report.

## Disclosure

The authors have nothing to report.

## Ethics Statement

This study was approved by the Institutional review boards at our institutions.

## Data Availability

The data that support the findings of this study are available on request from the corresponding author. The data are not publicly available due to privacy or ethical restrictions.
